# Clade, Country and Region-specific HIV-1 Vaccines: Are they necessary?

**DOI:** 10.1186/1742-6405-2-3

**Published:** 2005-04-28

**Authors:** Karen S Slobod, Chris Coleclough, Scott A Brown, John Stambas, Xiaoyan Zhan, Sherri Surman, Bart G Jones, Amy Zirkel, Pamela J Freiden, Brita Brown, Robert Sealy, Mattia Bonsignori, Julia L Hurwitz

**Affiliations:** 1 Department of Infectious Diseases, St Jude Children's Research Hospital, 332 N. Lauderdale, Memphis, TN 38105 USA; 2 Department of Immunology, St Jude Children's Research Hospital, 332 N. Lauderdale, Memphis, TN 38105 USA; 3 Department of Pediatrics, College of Medicine, 899 Madison Ave., University of Tennessee, Memphis, TN 38163 USA; 4 Department of Pathology, College of Medicine, 899 Madison Ave., University of Tennessee, Memphis, TN 38163 USA; 5 Department of Microbiology and Immunology, University of Melbourne, Vic 3010, Australia; 6 Department of Clinical and Biological Sciences, University of Insubria, Varese, 21100, Italy

## Abstract

Today, scientists are often encouraged to custom-design vaccines based on a particular country or clade. Here, we review the scientific literature and then suggest that the overwhelming endeavor to produce a unique vaccine for every world region or virus subtype may not be necessary.

## Clade, country or region-specific vaccines

It is generally agreed that HIV-1 arose decades ago by transfer of virus from chimps to humans [1]. The subsequent travel of infected persons and the continued practice of high-risk behaviors fostered virus transmission to virtually every world region. Once HIV-1 awareness was heightened and HIV-1 sequencing projects were implemented, regional similarities of viral sequences, presumably a consequence of the founder effect, became evident. Clade designations (e.g. clade A, B, C) were then used as a means to categorize viruses based on genetic sequence; thus such clade designations also tended to cluster viruses according to geographical location. Today, due to continuous virus transmission, mutation and recombination, the demarcation of HIV-1 subtypes has become increasingly blurred, and the categorization of viruses by clade is increasingly difficult [2-5]. Nonetheless scientists are currently encouraged to custom-design vaccines based on a particular country or clade [6-11]. To this end, a single viral sequence may be selected, possibly based on a formula of ancestry or consensus, to represent all other viruses in the targeted category.

Designing vaccines in this way prompts careful consideration: must a unique vaccine be prepared to represent every clade, country or region of the world? If so, how will this be accomplished and for which country should first vaccines be produced? Who will decide? The complexity of such an undertaking and the many difficulties that attend it encourage a second look at the strategy. Review of the scientific literature may provide reassurance that the seemingly unachievable endeavor to custom-produce a vaccine for every clade, country or region may not be necessary.

## Do immune responses discriminate between clades?

While differences in encoded protein sequence may permit discrimination between certain HIV-1 subtypes, successful vaccine development requires that viral proteins elicit protective immune responses, regardless of sequence. It has long been known that clades, as defined by genetic sequence, do not correspond to immunotypes, as defined by mutually exclusive immune responses [12-14]. Both B- and T-cells elicited by a virus from one clade may recognize viruses from other clades. This cross-clade responsiveness is explained by the fact that the B- and T-cells recognize precise epitopes rather than the overall sequence similarity of viruses. Antibody binding depends on three-dimensional structure, and the molecular structures bound by antibodies can occur on proteins that differ widely in primary sequence. T-cells recognize peptides in association with Class I or Class II MHC molecules, but like B-cells, T-cells can cross-react with non-identical targets. Conversely, two viruses may have 99% sequence similarity, yet a particular neutralizing antibody or T-cell receptor may discriminate between them. This discrimination may be due to a single amino acid change within the receptor contact site or in a sequence that alters epitope display [15,16]. Thus it is the detail of epitope and epitope context, not overall sequence similarity that defines lymphocyte specificity.

## Cross-clade protection is achieved by priming the immune system with diverse viral sequences from a single clade

The issues described above suggest that although a single-component vaccine may not be sufficient to target *any *clade, a cocktail vaccine, designed to represent the natural diversity of HIV-1, may be sufficient to target *all *clades. The latter point is supported by studies of HIV-1-infected humans and SIV-infected macaques. Although infected subjects cannot clear endogenous virus (due to its sequestration in "privileged" sites, hidden from the immune system), most individuals are resistant to super-infection [17-22]. This protection likely arises as the result of many successive rounds of endogenous viral mutation in the infected host. Each time an immune response is elicited in the periphery of an infected subject, new virus mutants appear [23,24]. The new viruses, by definition, have altered T- and B-cell determinants, allowing escape from the established antibodies and T-cell receptors. Following several rounds of immune response and virus escape, the B- and T-cells are primed to recognize a broad spectrum of determinants [25]. Thus, superinfections are rare, even in subjects likely to have been serially exposed to viruses from different clades. The rare double infections in humans (explaining the origin of virus recombinants [4]) are perhaps a consequence of (i) drug regimens which block the natural evolution of virus in the infected subject, (ii) repeated HIV-1 exposures prior to maturation of the adaptive immune response, and/or (iii) disease-related immunodeficiency.

The fact that a mature immune response to HIV-1 cannot clear sequestered virus, but can prevent super-infection emphasizes the importance of priming the system preemptively. Similar considerations pertain to the design of vaccines against human herpesviruses (e.g. VZV and EBV), as these viruses provoke both lifelong infections and long-term protective immunity to superinfection. As with the successful VZV vaccine [26], an effective HIV-1 vaccine should be administered before virus exposure, infection and sequestration.

## Could a cocktail vaccine ever be large enough to prevent HIV-1 infections?

Perhaps careful vaccine formulation will preclude the need for assembly of enormous cocktails. Consideration that envelope structure is constrained by function suggests that the formulation of an effective envelope-based vaccine is feasible. The virus envelope must bind target cells to mediate infection, and only a few target cell receptor molecules (e.g. CD4, CCR5, CXCR4), have been described. Therefore, the number of discrete envelope shapes that maintain full cell-binding potential and function is likely to be limited [27]. Because the virus envelope is the target of both neutralizing antibodies and T cells, the strengths of both arms of the immune system may be harnessed by an envelope-based vaccine cocktail [28-30]. Diverse proteins need not be cross-inhibitory. In fact, type-specific immune responses have been recognized toward a single envelope construct represented as only 1% of a mixed vaccine [31]. Cocktail vaccines are effective in controlling other diverse pathogens (e.g. pneumococcus, poliovirus), despite early doubts about their prospect of success [32].

## Clade, Country or Region-specific HIV Vaccines may not be necessary

The assembly of envelope cocktail vaccines will probably be necessary to represent the natural diversity of HIV-1, even within a single clade. Careful vaccine design may reveal a cocktail formulation able to prevent virus infections in every world region, and to overcome the political and financial dilemmas associated with the production of clade, country or region-specific vaccines.

## References

[B1] Perrin L, Kaiser L, Yerly S (2003). Travel and the spread of HIV-1 genetic variants. Lancet Infect Dis.

[B2] Delwart EL, Orton S, Parekh B, Dobbs T, Clark K, Busch MP (2003). Two percent of HIV-positive U.S. blood donors are infected with non-subtype B strains. AIDS Res Hum Retroviruses.

[B3] Anderson JP, Rodrigo AG, Learn GH, Madan A, Delahunty C, Coon M (2000). Testing the hypothesis of a recombinant origin of human immunodeficiency virus type 1 subtype E. J Virol.

[B4] McClutchan FE, Carr JK, Murphy D, Piyasirisilp S, Gao F, Hahn B (2002). Precise mapping of recombination breakpoints suggests a common parent of two BC recombinant HIV type 1 strains circulating in China. AIDS Res Hum Retroviruses.

[B5] Thomson MM, Perez-Alvarez L, Najera R (2002). Molecular epidemiology of HIV-1 genetic forms and its significance for vaccine development and therapy. Lancet Infect Dis.

[B6] Williamson C, Morris L, Maughan MF, Ping LH, Dryga SA, Thomas R (2003). Characterization and selection of HIV-1 subtype C isolates for use in vaccine development. AIDS Res Hum Retroviruses.

[B7] Gaschen B, Taylor J, Yusim K, Foley B, Gao F, Lang D (2002). Diversity considerations in HIV-1 vaccine selection. Science.

[B8] Hanke T, Barnfield C, Wee EG, Agren L, Samuel RV, Larke N (2003). Construction and immunogenicity in a prime-boost regimen of a Semliki Forest virus-vectored experimental HIV clade A vaccine. J Gen Virol.

[B9] Novitsky V, Smith UR, Gilbert P, McLane MF, Chigwedere P, Williamson C (2002). Human immunodeficiency virus type 1 subtype C molecular phylogeny: consensus sequence for an AIDS vaccine design?. J Virol.

[B10] Hanke T, McMichael AJ (2000). Design and construction of an experimental HIV-1 vaccine for a year-2000 clinical trial in Kenya. Nature Medicine.

[B11] Agwale SM, Zeh C, Robbins KE, Odama L, Saekhou A, Edubio A (2002). Molecular surveillance of HIV-1 field strains in Nigeria in preparation for vaccine trials. Vaccine.

[B12] Cao H, Mani I, Vincent R, Mugerwa R, Mugyenyi P, Kanki P (2000). Cellular immunity to human immunodeficiency virus type 1 (HIV-1) clades: relevance to HIV-1 vaccine trials in Uganda. J Infect Dis.

[B13] Moore JP, Cao Y, Leu J, Qin L, Korber B, Ho DD (1996). Inter- and intraclade neutralization of human immunodeficiency virus type 1: genetic clades do not correspond to neutralization serotypes but partially correspond to gp120 antigenic serotypes. J Virol.

[B14] Ferrantelli F, Kitabwalla M, Rasmussen RA, Cao C, Chou TC, Katinger H (2004). Potent cross-group neutralization of primary human immunodeficiency virus isolates with monoclonal antibodies – implications for acquired immunodeficiency syndrome vaccine. J Infect Dis.

[B15] D'Costa S, Slobod KS, Webster RG, White SW, Hurwitz JL (2001). Structural features of HIV envelope defined by antibody escape mutant analysis. AIDS Res Hum Retroviruses.

[B16] Zhan X, Slobod KS, Surman S, Brown SA, Lockey TD, Coleclough C (2003). Limited breadth of a T-helper cell response to a human immunodeficiency virus envelope protein. J Virol.

[B17] Gonzales MJ, Delwart E, Rhee SY, Tsui R, Zolopa AR, Taylor J (2003). Lack of detectable human immunodeficiency virus type 1 superinfection during 1072 person-years of observation. J Infect Dis.

[B18] Tsui R, Herring BL, Barbour JD, Grant RM, Bacchetti P, Kral A (2004). Human immunodeficiency virus type 1 superinfection was not detected following 215 years of injection drug user exposure. J Virol.

[B19] Altfeld M, Allen TM, Yu XG, Johnston MN, Agrawal D, Korber BT (2002). HIV-1 superinfection despite broad CD8+ T-cell responses containing replication of the primary virus. Nature.

[B20] Daniel MD, Kirchhoff F, Czajak SC, Sehgal PK, Desrosiers RC (1992). Protective effects of a live attenuated SIV vaccine with a deletion in the nef gene. Science.

[B21] Burns DPW, Desrosiers RC (1994). Envelope sequence variation, neutralizing antibodies and primate lentivirus persistence. Curr Top Microbiol Immunol.

[B22] Titti F, Sernicola L, Geraci A, Panzini G, Di Fabio S, Belli R (1997). Live attenuated simian immunodeficiency virus prevents super-infection by cloned SIVmac251 in cynomolgus monkeys. J Gen Virol.

[B23] Wrin T, Crawford L, Sawyer L, Weber P, Sheppard HW, Hanson CV (1994). Neutralizing antibody responses to autologous and heterologous isolates of human immunodeficiency virus. J Acquir Immune Defic Syndr.

[B24] Richman DD, Wrin T, Little SJ, Petropoulos CJ (2003). Rapid evolution of the neutralizing antibody response to HIV type 1 infection. Proc Natl Acad Sci U S A.

[B25] Rencher SD, Slobod KS, Dawson D, Lockey TD, Hurwitz JL (1995). Does the key to a successful HIV vaccine lie among the envelope sequences of infected individuals?. Aids Res Hum Retroviruses.

[B26] Seward JF, Watson BM, Peterson CL, Mascola L, Pelosi JW, Zhang JX (2002). Varicella disease after introduction of varicella vaccine in the United States, 1995–2000. JAMA.

[B27] Nyambi PN, Nadas A, Mbah HA, Burda S, Williams C, Gorny MK (2000). Immunoreactivity of intact virions of human immunodeficiency virus type 1 (HIV-1) reveals the existence of fewer HIV-1 immunotypes than genotypes. J Virol.

[B28] Slobod KS, Lockey TD, Howlett N, Srinivas RV, Rencher SD, Freiden PJ (2004). Subcutaneous administration of a recombinant vaccinia virus vaccine expressing multiple envelopes of HIV-1. Eur J Clin Microbiol Infect Dis.

[B29] Hurwitz JL, Slobod KS, Lockey TD, Wang S, Chou T-HW, Lu S (2005). Application of the polyvalent approach to HIV-1 vaccine development. Current Drug Targets-Infectious Disorders.

[B30] Stambas J, Brown SA, Gutierrez A, Sealy R, Yue W, Jones B (2005). Long lived multi-isotype anti-HIV antibody responses following a prime-double boost immunization strategy. Vaccine.

[B31] Zhan X, Slobod KS, Surman S, Brown SA, Coleclough C, Hurwitz JL (2004). Minor components of a multi-envelope HIV vaccine are recognized by type-specific T-helper cells. Vaccine.

[B32] Burnet FM (1945). Poliomyelitis in the light of recent experimental work. Health Bulletin Department of Health-Victoria, Australia.

